# Osseous Metastases in Thyroid Cancer: Unveiling Risk Factors, Disease Outcomes, and Treatment Impact

**DOI:** 10.3390/cancers15143557

**Published:** 2023-07-10

**Authors:** Zenat Ahmed Khired, Mohammad H. Hussein, Jessan A. Jishu, Ahmed A. Toreih, Aly A. M. Shaalan, Mohammed M. Ismail, Manal S. Fawzy, Eman A. Toraih

**Affiliations:** 1 Department of Surgery, College of Medicine, Jazan University, Jazan 45142, Saudi Arabia; zkherd@jazanu.edu.sa; 2 Division of Endocrine and Oncologic Surgery, Department of Surgery, School of Medicine, Tulane University, New Orleans, LA 70112, USA; mhussein1@tulane.edu; 3 School of Medicine, Tulane University, New Orleans, LA 70112, USA; jjishu@tulane.edu; 4 Department of Orthopedic Surgery, Faculty of Medicine, Suez Canal University, Ismailia 41522, Egypt; toreih@hotmail.com; 5 Department of Anatomy, Faculty of Medicine, Jazan University, Jazan 45142, Saudi Arabia; alyshaalan@yahoo.com; 6 Department of Histology and Cell Biology, Faculty of Medicine, Suez Canal University, Ismailia 41522, Egypt; 7 Department of Anatomy, Faculty of Medicine, Northern Border University, Arar 91431, Saudi Arabia; 2351357609@nbu.edu.sa; 8 Department of Medical Biochemistry and Molecular Biology, Faculty of Medicine, Suez Canal University, Ismailia 41522, Egypt; 9 Department of Biochemistry, Faculty of Medicine, Northern Border University, Arar 91431, Saudi Arabia; 10 Genetics Unit, Department of Histology and Cell Biology, Suez Canal University, Ismailia 41522, Egypt

**Keywords:** thyroid cancer, bone, osseous, metastasis, risk factor, SEER

## Abstract

**Simple Summary:**

Thyroid cancer is the most common endocrine cancer and is becoming increasingly prevalent. Although its prognosis is generally favorable, bone metastasis is a notable complication that significantly decreases survival rates. Currently, there is no definitive cure as most treatments are palliative to relieve patients of any pain or other symptoms. We assessed the incidence and influence of bone metastasis on thyroid cancer patients to understand the risk factors and outcomes which can improve clinical decision making and research endeavors.

**Abstract:**

Bone is the second most common site of metastasis in patients with thyroid cancer (TC) and dramatically impacts overall survival and quality of life with no definitive cure, yet there is no extensive study of the demographic and clinical risk factors in the recent literature. Data regarding 120,754 TC patients with bone metastasis were retrieved from the Surveillance, Epidemiology, and End Results (SEER) database. Univariate and multivariate analyses were used to identify the risk factors of bone metastasis occurring in various histologies of TC. Cox regression was performed to analyze the influence of bone metastasis on overall survival. Hazard ratios were computed to analyze the association between bone metastasis and the primary outcomes. Of the 120,754 records collected from the SEER database from 2000 to 2019, 976 (0.8%) presented with bone metastasis, with occurrence being the greatest in patients of age ≥ 55 years (OR = 5.63, 95%CI = 4.72–6.71), males (OR = 2.60, 95%CI = 2.27–2.97), Blacks (OR = 2.38, 95%CI = 1.95–2.9) and Asian or Pacific Islanders (OR = 1.90, 95%CI = 1.58–2.27), and single marital status. TC patients presenting with bone metastasis (HR = 2.78, 95%CI = 2.34–3.3) or concurrent bone and brain metastases (HR = 1.62, 95%CI = 1.03–2.55) had a higher mortality risk. Older age, gender, race, and single marital status were associated with bone metastasis and poorer prognosis in TC patients at initial diagnosis. Understanding such risk factors can potentially assist clinicians in making early diagnoses and personalized treatment plans, as well as researchers in developing more therapeutic protocols.

## 1. Introduction

Thyroid cancer (TC) is a neoplasm that originates from the follicular cells of the thyroid gland, representing the most prevalent endocrine malignancy. In recent decades, there has been a significant global increase in the incidence of TC, making it the fastest-growing cancer type in both men and women [1]. Despite this growth in incidence, TC generally has a favorable prognosis, with an overall five-year survival rate exceeding 98% [2]. Impressive prognosis can be attributed to active surveillance and management of TC patients, but the recent literature has shed light on the fact that survival rates drop significantly in the context of distant metastases, with the bone being one of the most prevalent sites [3]. Bone metastases are a noteworthy complication of TC, significantly impacting patient outcomes and overall quality of life. Identifying the risk factors and clinical implications of bone metastases is crucial for optimizing patient management and enhancing therapeutic strategies [4].

Bone metastases can develop in various TC histologies. According to the literature, such metastases occur in roughly 7–28% of follicular (FTC), 1.4–7% of papillary (PTC), and 16–19% of medullary (MTC) TC patients during follow-up [4]. Its presence in differentiated thyroid cancer (DTC) patients can decrease survival, with 1-, 2, 3-, 5-, and 10-year survival rates being 93%, 89%, 80%, 61%, and 27%, respectively, from the first diagnosis of bone metastases [5]. The decline in survival rates can also be attested to bone metastases concurrently presenting in other regions. A recent population-based study of 64,083 TC patients revealed that TC patients presenting only with bone metastases were associated with a longer survival time (66 months) than those with metastases to bone along with a single (14 months) or multiple (6 months) extraosseous regions [6]. There exist effective treatments for extraosseous metastases, including multikinase inhibitors, ^131^I therapy [7], or complete metastasectomy [8] for pulmonary or brain metastases, but limited treatment for bone metastases as the only certified one that currently exists is palliative for symptomatic relief. Wide osseous resection can be an option for some patients, but recent studies note significant disadvantages such as infections, dislocation complications, and anatomical manipulations [9].

This prompts the need to identify risk factors predicting bone metastases in TC patients, as early detection and treatment entail prolonged survival and better patient outcomes [10]. Recently, a nomogram was developed to identify high-risk TC patients [11] but did not extensively consider demographic characteristics, such as marital status or income levels. Other recent studies successfully confirmed the influence of select clinical and demographic characteristics on bone metastases but did not find an impact of demographic factors on overall and TC-specific survival [6].

Our study aims to conduct a comprehensive, population-based analysis of bone metastases in TC, intending to decipher the multifaceted layers of its epidemiological characteristics, risk factors, disease outcomes, and the consequential impact of diverse treatment modalities. Such an analysis will manifest into actionable insights, shedding light on the prevalence, geospatial distribution, and characteristic patterns of bone involvement in TC, which will subsequently direct us toward more strategic prevention and management strategies. Understanding and identifying risk factors will provide a robust platform for risk stratification, aiding in the implementation of personalized treatment plans designed to meet the unique needs and characteristics of individual patients. Furthermore, by meticulously assessing disease outcomes, we seek to offer invaluable prognostic information that will be instrumental in refining treatment decision-making processes. Moreover, evaluating the effects of different treatment modalities is expected to significantly influence clinical practices, elevating the standards of patient care by integrating the most efficient and effective treatment methods. Ultimately, our study enhances the current understanding of the burden of bone metastases in TC. By offering precise and data-driven treatment strategies, we aim to usher in a new era of improved patient outcomes, mitigating the debilitating impact of this disease.

## 2. Materials and Methods

### 2.1. Data Source

We analyzed a retrospective cohort study using “The Surveillance, Epidemiology, and End Results (SEER)” database (2000–2019), with data combined from registries 17 and 22. SEER is a comprehensive source of population-based cancer data in the United States. SEER collects and publishes cancer incidence, prevalence, survival, and mortality data from population-based cancer registries that cover approximately 34.6% of the US population. The database is maintained by the National Cancer Institute (NCI) and is widely used by researchers, clinicians, and policymakers to study cancer trends, outcomes, and disparities over time. This approach offers the advantage of capturing a diverse range of patient demographics and clinical characteristics, which can provide valuable insights into the real-world implications of osseous metastases in TC.

### 2.2. Study Population

Participants were eligible for the study if they were individuals of any age, sex, and race; diagnosed with TC as their first primary tumor; and had bone metastases at the time of diagnosis. Patients were excluded from the study if they had metastasis exclusively in other sites rather than bone or had a history of prior primary cancer before the diagnosis of TC.

### 2.3. Study Variables

The study variables for our retrospective cohort analysis include a range of demographic, clinical, and treatment-related factors. Demographic variables include age, gender, race, ethnicity, marital status, metropolitan residence status, and annual household income. Clinical factors encompass histological type, T staging (primary tumor size and extent), N staging (lymph node involvement), extension to nearby structures, and management strategies such as cancer-directed surgery, radiotherapy, radioactive iodine, and systemic therapy. 

### 2.4. Primary Outcomes

The primary outcomes assessed in this study are overall survival (OS), TC-specific survival (TCSS), recurrence, and subsequent second primary cancer in patients with osseous metastases at diagnosis. These outcomes help to determine the prognostic factors and real-world implications of bone metastases in TC, which can inform clinical decision making and treatment strategies. 

### 2.5. Statistical Analysis

Descriptive statistics were used to summarize the demographic and clinical characteristics of the study population, including frequencies and percentages for categorical variables and means, medians, and standard deviations for continuous variables. The R packages were used for analysis. Univariate and multivariate analyses were performed to identify potential risk factors associated with osseous metastases. For categorical variables, chi-square or Fisher’s exact tests were used, whereas continuous variables were assessed using the independent t-test or Mann–Whitney U test, depending on the normality of the data distribution. Cox proportional hazards regression models were utilized to assess the impact of osseous metastases on survival outcomes, adjusting for potentially confounding variables such as age, sex, tumor size, histologic subtype, and other relevant factors. Hazard ratios (HRs) with corresponding 95% confidence intervals (CIs) were calculated to quantify the association between osseous metastases and the primary outcomes. Kaplan–Meier survival curves were generated, and the log-rank test was used to compare group differences. A *p*-value of less than 0.05 was considered statistically significant for all tests.

## 3. Results

### 3.1. Characteristics of the Study Population

**Figure 1:**
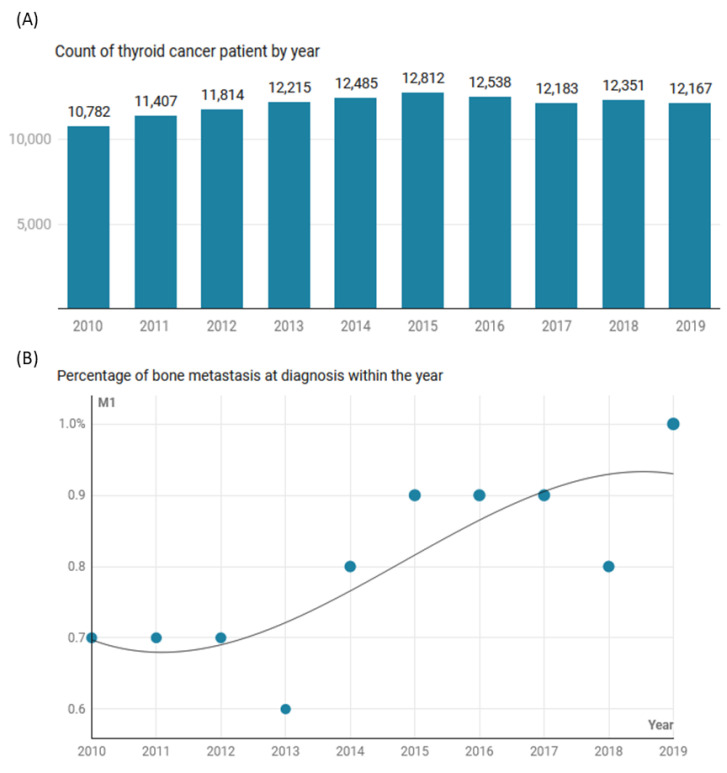
The trend in thyroid cancer in SEER registries 17 and 22 (2000–2019). (**A**) Count of patients per year. (**B**) Percentage of bone metastasis within each year.

The study analyzed 120,754 records with TC, according to the eligibility criteria. These included 119,778 patients in the M0 stage and 976 (0.8%) patients presenting with bone metastases. Across the studied years, there was generally a higher rate of patients with bone metastases detected at TC presentation (Figure 1).

**Figure 2:**
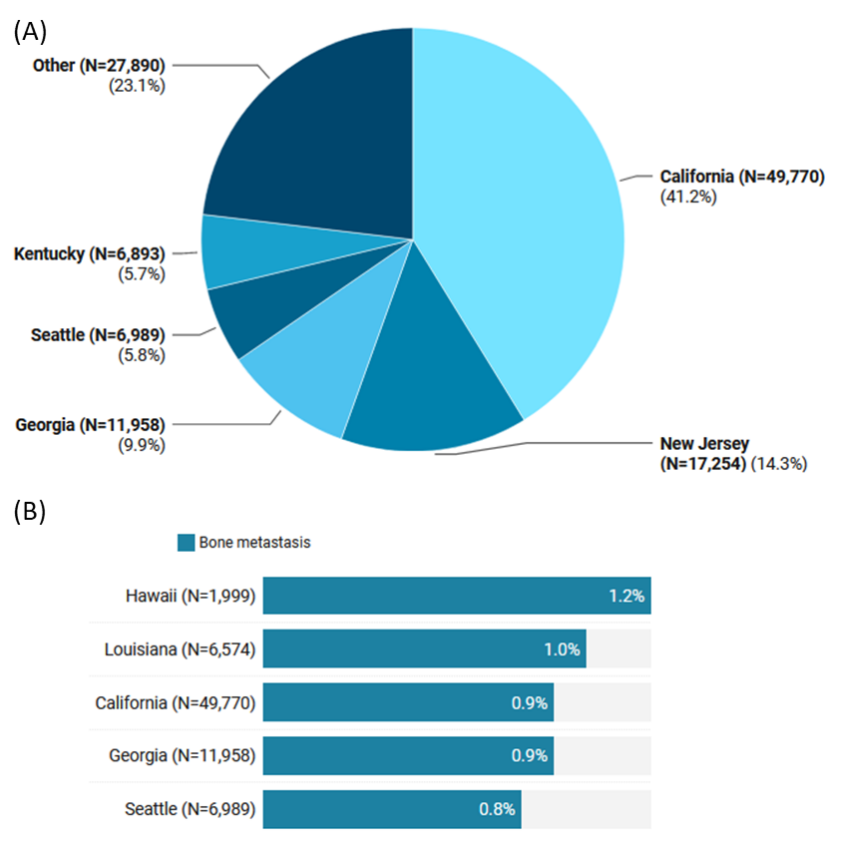
Geographical distribution of thyroid cancer patients in SEER database (2000–2019). (**A**) Distribution of cases according to the US States. (**B**) Percentage of bone metastasis within the State. The top 5 States are only shown.

The geographical distribution of TC cases across the US states was predominantly in California (41.2%), followed by New Jersey (14.3%), Georgia (9.9%), and Seattle (58%). However, the highest proportion of bone metastases detected at diagnosis was found in Hawaii (1.2% out of 1999 patients) and Louisiana (1% of 6574 patients) (Figure 2). 

A comparison between TC patients with and without bone metastases is presented in Table 1. Patients who presented with bone metastases were more likely to be older than 55 years (82.2% vs. 40%, *p* < 0.001), males (47.1% vs. 24.6%, *p* < 0.001), Black (13.3% vs. 6.6%, *p* < 0.001), and widowed (14.6% vs. 5.1%, *p* < 0.001).

Papillary TC (PTC) was the most common (N = 107,227) histology, accounting for 88.8% of the study population, whereas follicular (FTC), anaplastic (ATC), and medullary (MTC) TC represented 4.7% (N = 5718), 0.5% (N = 649), and 1.7% (N = 1993), respectively. The bone metastases group was more likely to have advanced T stage (T3/4: 51.8% vs. 17.8%, *p* < 0.001) and lymph node metastasis (45.6% vs. 24.1%, *p* < 0.001) (Table 2).

**Figure 3:**
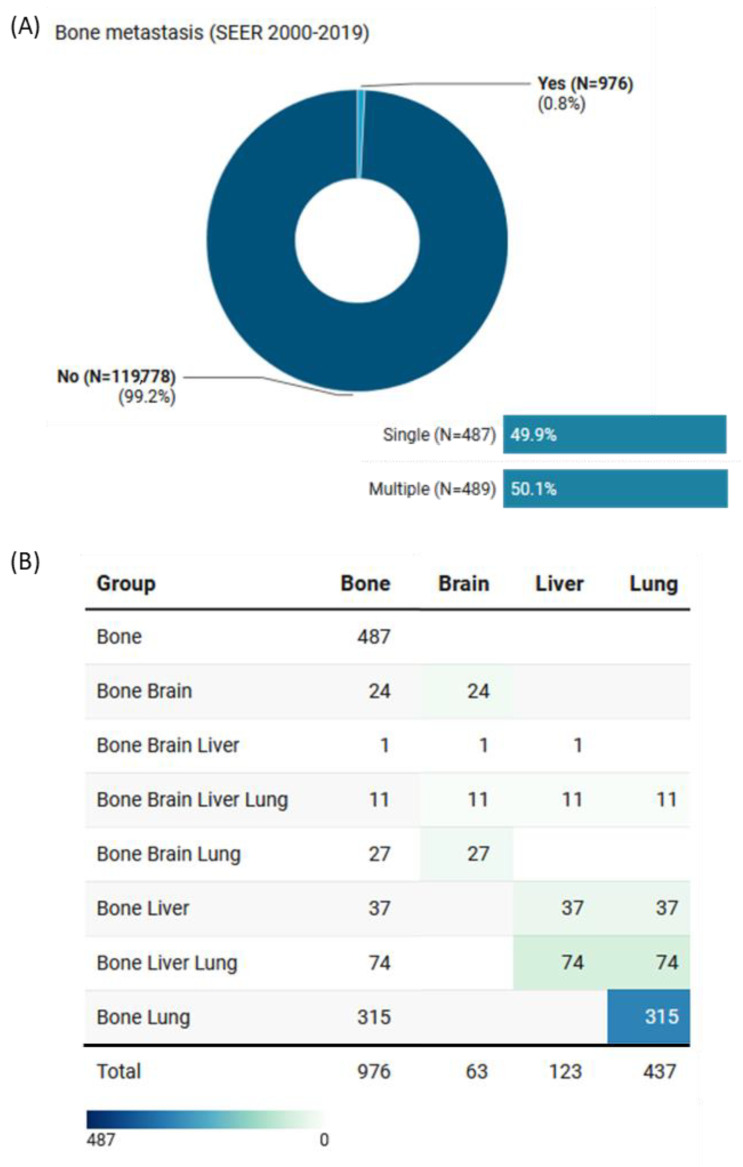
Prevalence of bone metastasis in the study population (SEER database: 2000–2019). (**A**) Frequency of bone metastasis in non-medullary thyroid cancer patients. Of those with bone metastasis, 50.1% had concomitant metastasis in either brain, liver, or lung. (**B**) Distribution of patients with bone metastasis associated with involvement of other organs.

Of the 976 (0.8%) who presented with bone metastases, 489 (51.3%) had concomitant metastases in other organs. These included 63 cases with brain metastases, 123 with liver metastases, and 437 with lung metastases (Figure 3).

### 3.2. Risk Factors Predicting Bone Metastasis at the Time of Diagnosis

**Figure 4:**
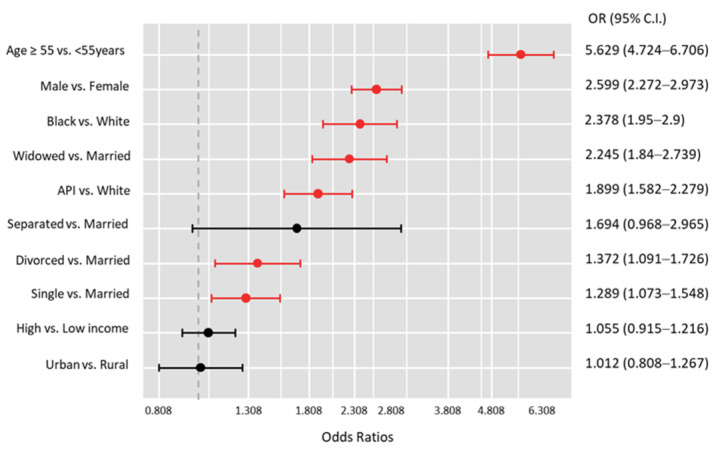
Predictor risk factors for having bone metastasis at the time of diagnosis of thyroid cancer. Binary logistic regression analysis was employed. Odds ratio (OR) and 95% confidence intervals (CI) were reported for each variable. Red bars indicate a significant association. API: Asian or Pacific Islander.

Multivariate analysis of patient characteristics with and without bone metastases at the time of diagnosis revealed that patients with bone metastases were more likely to be males (OR = 2.60, 95%CI = 2.27–2.97, *p* < 0.001), older than 55 years (OR = 5.63, 95%CI = 4.72–6.71, *p* < 0.001), Black (OR = 2.38, 95%CI = 1.95–2.9, *p* < 0.001) or Asian or Pacific Islander (OR = 1.90, 95%CI = 1.58–2.27, *p* < 0.001), and less likely to be married (all *p* < 0.05) (Figure 4).

### 3.3. Disease Outcomes

The analysis of disease outcomes revealed that among 400 TC patients who experienced recurrence, recurrence rates did not differ significantly between groups (*p* = 0.89). However, 22,073 patients (18.3%) developed second primary malignancies, and those with bone metastases had a significantly higher incidence of developing these secondary cancers (22.7% vs. 18.2%, *p* < 0.001) (Table 3).

Table 4 and Table 5 present the results of the multivariate regression analysis, revealing several risk factors associated with the development of second primary malignancies following TC diagnosis.

Table 4 shows that patients of age ≥ 55 years, regardless of bone metastases, had a significantly higher risk of developing second primary cancers (no bone metastases: OR = 3.91, *p* < 0.001; bone metastases: OR = 3.64, *p* = 0.001). Male patients without bone metastases had a higher risk (OR = 1.32, *p* < 0.001), while there was no significant association for male patients with bone metastases (OR = 1.09, *p* = 0.71). Regarding race, Asian or Pacific Islander (API) patients with bone metastases had a lower risk than White patients (OR = 0.46, *p* = 0.047). Patients who underwent surgery for their TC without bone metastases were less likely to develop second primary cancers (OR = 0.41, *p* < 0.001), while the opposite was true for those with bone metastases (OR = 2.45, *p* = 0.008).

### 3.4. Survival Analysis

**Figure 5:**
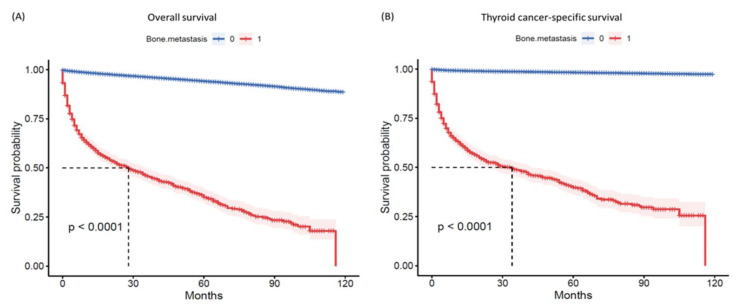
Survival analysis. (**A**) Kaplan–Meier survival curve for overall survival. (**B**) Kaplan–Meier survival curve for thyroid cancer-specific survival. The log-rank test was used to compare patients with and without bone metastasis.

The overall mortality rate was 6.1% (N = 7419). Patients with bone metastases had a significantly lower survival rate of 41.7%, compared to 94.3% in the non-metastatic group (*p* < 0.001) (Table 3). The high mortality rate in the bone metastases group was primarily caused by TC (81.2%). The remaining deaths were attributed to other causes (18.8%), mainly heart and lung diseases. Additionally, survival times were significantly shorter in the metastases group, with a median of 1.17 years (IQR: 0.25–3.5) compared to 4.2 years (1.8–6.8) in the non-metastases group (*p* < 0.001) (Figure 5).

Table 6 presents the findings of a multivariate Cox regression analysis, which evaluated various risk factors concerning overall survival in TC patients. The findings reveal that patients with bone metastasis (HR = 2.78, 95%CI = 2.34–3.3, *p* < 0.001) or concomitant bone and brain metastases (HR = 1.62, 95%CI = 1.03–2.55, *p* = 0.035) had a higher risk of mortality. Advanced tumor size and nodal metastasis were also associated with an increased risk of mortality (*p* < 0.001), while an annual household income of ≥USD 75K (HR = 0.85, 95%CI = 0.80–0.90, *p* < 0.001) and residing in an urban area (HR = 0.82, 95%CI = 0.75–0.89, *p* < 0.001) were linked to a lower risk of mortality. 

Furthermore, the study found that different treatment modalities were associated with varying degrees of survival benefit. Patients who underwent surgical resection of primary tumors had an 80% decreased mortality risk (HR = 0.20, 95%CI = 0.18–0.22, *p* < 0.001). Those who received radiation therapy had a 34% decreased risk (HR = 0.66, 95%CI = 0.62–0.70, *p* < 0.001), while patients who received systematic therapy had a 9% decreased risk (HR = 0.91, 95%CI = 0.86–0.96, *p* = 0.001), as demonstrated in Table 6.

## 4. Discussion

Bone is the second most common location for metastasis in patients with TC. Such metastasis increases the occurrence of skeletal-related events (SREs) such as pathologic fractures and spinal cord compression, dramatically impacting quality of life and morbidity. Farooki et al. found that in a study of 245 DTC patients with bone metastasis, 78% developed a first SRE after a median of 5 months, and 65% of them developed a subsequent SRE after a median of 10.7 months following the first event, with 39% of all TC patients with bone metastasis developing three or more SREs [12]. Matta-Coelho et al. found that in a study of 86 DTC patients with bone metastasis, 76.7% developed SREs [13]. Treating early bone metastasis from DTC can reduce the number of SREs and improve the extent and quality of life. For example, Orita et al. found that in a study of 50 DTC patients with bone metastasis, 50% of those that did not undergo bisphosphonate therapy developed SREs, compared to a remarkable 14% of those that did [14]. Early detection and treatment can also increase the chances of finding a cure [15].

In this study, a sizable cohort of 120,754 TC patients was examined, and it was discovered that 0.8% of cases presented with bone metastasis at the time of diagnosis, comparable to 0.36% as recently reported by Qi et al. [16], 0.5% as recently reported by Zhang et al. [6], and 0.97% as recently reported by Liu et al. [17]. To our knowledge, this study scrutinizes the largest sample size of DTC (and TC in general) patients with bone metastasis. The large sample size can be attributed to data gathered over 20 years, which is more significant than most studies, improved diagnostic methods, and various management protocols between states. With this study investigating a large population with all TC histologies, such demographic and clinical findings are generalized and can provide helpful insight to clinicians in determining their decision making.

Patients who had bone metastasis were more likely to be over the age of 55, male, Black or Asian or Pacific Islander, or single (never married, separated, divorced, or widowed). Recent studies corroborate the association of older age [6,11,17,18], male gender [6,19], and black race of TC patients [6,11] with bone metastasis. Of these factors, older age is the most supported in the literature. It may be worth examining if the drawbacks behind wide osseous resection are solely due to the older population, which is much more at risk of bone metastasis, or if they apply to a younger population. This study illuminates Asian or Pacific Islander races and single status as new characteristics of risk of bone metastasis. Moreover, these patients had a higher frequency of advanced T-stage and lymph node metastasis, corroborated by recent literature [16].

The present study also found that patients recorded in Hawaii and Louisiana had the most significant proportion of bone metastasis detected at diagnosis. Multiple reasons can account for this, including but not limited to a higher density of endocrinologists and orthopedic physicians, explaining increased detection and diagnosis; lower socioeconomic status, implying delayed diagnosis and treatment of TC allowing for metastasis; or environmental factors contributing to this observation. However, this finding warrants further study.

It is well known that bone metastases are present more commonly in aggressive thyroid cancers, such as ATC. Interestingly, the findings revealed that PTC was the most prevalent of all histologies that involved bone metastases, comprising 37.8% of all cases, in contrast to ATC, which comprised 23.7% of all cases. One straightforward explanation for this includes the much larger PTC patients, composed of 88.8% of the present study’s population. Several studies acknowledge this notion and justify that bone metastasis will indeed be higher in PTC cases compared to other TC histologies since there is a larger population in that cohort [20,21]. Since the present study found that ATC patients were more likely to develop bone metastases than PTC patients (OR = 1.32, *p* = 0.47), the composition of patients can likely justify the finding of higher bone metastases in PTC as well as the statistically insignificant finding of ATC patients being more at risk. Patient population and referral were also used to potentially justify discrepancies between Wu et al. and Marcocci et al. regarding varying bone metastases rates in PTC [22,23]. Nevertheless, literature within the past two decades has discussed the difficulty of identifying the origin of bone metastasis despite diagnostic advances and has suggested considering the possibility of underlying lesions [23].

Patients with bone metastasis had a markedly lower survival rate of 41.7%, compared to 94.3% in the non-metastatic group. Multivariate analysis revealed that patients with bone metastasis or concomitant bone and brain metastases had a higher risk of mortality, while advanced tumor size and nodal metastasis were also associated with an increased risk of mortality. The study also found that different treatment modalities were associated with varying degrees of survival benefit, with surgical resection of primary tumors associated with an 80% decreased mortality risk.

The present study’s findings on risk factors predicting bone metastasis at diagnosis are essential for clinicians in identifying patients at higher risk and tailoring treatment plans accordingly. Such prediction as early as the diagnosis has high clinical value as it can prevent metachronous bone metastasis, as Mazziotti et al. [24] recently found that it shows very aggressive clinical behavior with a significant association for developing SREs and can influence survival. Although bone metastasis occurs more frequently in other TC histologies than in PTC, the present study shows a more significant number of patients who undergo bone metastases. This finding is supported by Wexler, who advocates for screening PTC patients for extra-cervical metastases due to its high prevalence [21]. Screening is essential, as a previous study has demonstrated that a late diagnosis of bone metastases is associated with a poorer prognosis than early detection [25]. The timely identification of bone metastases is crucial for implementing appropriate interventions to prevent or delay SREs, such as bisphosphonates which induce osteoclast apoptosis and improve patient outcomes [26]. To facilitate early detection, recommended screening methods for bone metastases in TC encompass various imaging techniques. These may include bone scans, which utilize radioactive tracers to visualize areas of abnormal bone metabolism and identify metastatic lesions.

Additionally, positron emission tomography (PET), computed tomography (CT), and magnetic resonance imaging (MRI) can provide valuable insights into the presence and extent of bone metastasis. These imaging modalities offer non-invasive approaches to assess bone involvement in TC patients and play a crucial role in the early detection and monitoring of metastatic lesions. In conjunction with imaging modalities, regular follow-up appointments and thorough clinical evaluations are essential to effective screening for bone metastasis in TC. Healthcare providers should maintain a high level of vigilance and carefully monitor patients for any signs or symptoms that may indicate the presence of bone metastases, such as bone pain, fractures, or elevated levels of serum markers like alkaline phosphatase. Combining clinical evaluation with appropriate imaging techniques enhances the likelihood of early detection and enables prompt intervention. Our study uniquely emphasizes the importance of screening for bone metastases. Additionally, it highlights the high incidence of second primary malignancies in patients with bone metastasis and the significant benefits of various treatment options for these patients.

Although this population-based study examined the largest TC patient cohort with bone metastasis to date, certain limitations should be acknowledged. Firstly, it is essential to note that the data utilized in this study were obtained from the Surveillance, Epidemiology, and End Results (SEER) database, which primarily represents TC patients in the United States. Therefore, caution must be exercised when generalizing the findings to other populations or regions, as variations in demographics, healthcare systems, and genetic profiles may exist. The applicability of the results to different populations outside the United States should be further investigated in future studies with broader international representation. Moreover, while the SEER database is a comprehensive source of population-based cancer data in the United States, it has inherent limitations. It lacks certain subjective information, such as patient-reported outcomes, including bone pain or fatigue, which are essential factors in evaluating quality-of-life and patient-centered outcomes. The absence of these subjective measures may limit the comprehensive understanding of the impact of bone metastasis on patients’ well-being. Additionally, this study primarily focused on demographic and clinical risk factors associated with bone metastasis in TC patients without explicitly exploring the influence of genetic factors, such as mutations, that may coexist with the examined risk factors. Future research should consider integrating genetic analyses to elucidate the interplay between genetic predispositions and the identified risk factors to better understand the development and progression of bone metastasis in TC patients.

Overall, this study provides valuable insights into the characteristics of TC patients with bone metastasis, including their risk factors, the impact of treatment modalities, and disease outcomes. These findings have the potential to enhance patient care and management while identifying avenues for further research and the development of novel treatment options.

Understanding the epidemiology and impact of osseous metastases in TC is crucial for informing clinical decision making, prognostication, and the development of targeted therapeutic strategies. By advancing our knowledge of this complication, we aspire to improve the care and outcomes for patients at risk for or already affected by bone metastases in TC.

## 5. Conclusions

This study significantly contributes to the existing literature on TC by presenting the most extensive population-based analysis of bone metastasis to date, elucidating the associated demographic and clinical risk factors, and examining their impact on patient survival. The findings presented herein will serve as a foundational platform for future research endeavors and have the potential to shape the development of more effective diagnostic and therapeutic strategies for TC patients with bone metastases.

## Figures and Tables

**Table 1:** Demographic characteristics of the study population.

Characteristics	Levels	Total (N = 120,754)	No Metastasis (N = 119,778)	Bone Metastasis (N = 976)	*p*-Value
Age	<55 years	71,101 (58.9)	70,927 (59.2)	174 (17.8)	<0.001
	≥55 years	49,653 (41.1)	48,851 (40.8)	802 (82.2)	
Gender	Female	90,822 (75.2)	90,306 (75.4)	516 (52.9)	<0.001
	Male	29,932 (24.8)	29,472 (24.6)	460 (47.1)	
Race	White	96,233 (81)	95,546 (81.1)	687 (70.6)	<0.001
	Black	7921 (6.7)	7792 (6.6)	129 (13.3)	
	API	13,702 (11.5)	13,547 (11.5)	155 (15.9)	
	AI/AN	882 (0.7)	880 (0.7)	2 (0.2)	
Ethnicity	Not Hispanic/Latino	98,822 (81.8)	97,993 (81.8)	829 (84.9)	0.012
	Hispanic/Latino	21,932 (18.2)	21,785 (18.2)	147 (15.1)	
Marital Status	Married/Common law	71,142 (62.9)	70,610 (62.9)	532 (56.6)	<0.001
	Domestic Partner	501 (0.4)	497 (0.4)	4 (0.4)	
	Separated	1157 (1)	1144 (1)	13 (1.4)	
	Divorced	8428 (7.4)	8337 (7.4)	91 (9.7)	
	Widowed	5870 (5.2)	5733 (5.1)	137 (14.6)	
	Single (never married)	26,077 (23)	25,914 (23.1)	163 (17.3)	
Metropolitan	Metropolitan > 1M pop	73,699 (61.1)	73,094 (61.1)	605 (62.1)	0.53
	Metropolitan > 250K–1M	27,116 (22.5)	26901 (22.5)	215 (22.1)	
	Metropolitan of < 250K	8386 (7)	8324 (7)	62 (6.4)	
	Non-Metropolitan adj to a Metropolitan	6326 (5.2)	6267 (5.2)	59 (6.1)	
	Non-Metropolitan not adj to a Metropolitan	5072 (4.2)	5038 (4.2)	34 (3.5)	
Household Annual Income	USD 75,000+	40,527 (33.6)	40,190 (33.6)	337 (34.5)	0.72
	USD 70,000–USD 74,999	8542 (7.1)	8477 (7.1)	65 (6.7)	
	USD 65,000–USD 69,999	19,964 (16.5)	19,814 (16.5)	150 (15.4)	
	USD 60,000–USD 64,999	20,013 (16.6)	19,860 (16.6)	153 (15.7)	
	USD 55,000–USD 59,999	7741 (6.4)	7682 (6.4)	59 (6)	
	USD 50,000–USD 54,999	10,048 (8.3)	9965 (8.3)	83 (8.5)	
	USD 45,000–USD 49,999	5511 (4.6)	5455 (4.6)	56 (5.7)	
	USD 40,000–USD 44,999	4211 (3.5)	4173 (3.5)	38 (3.9)	
	USD 35,000–USD 39,999	2462 (2)	2438 (2)	24 (2.5)	
	<USD 35,000	1725 (1.4)	1714 (1.4)	11 (1.1)	

Data are presented as numbers and percentages. A two-sided chi-square test was used. Statistical significance was set at *p*-value < 0.05. API: Asian or Pacific Islander, AI/AN: Am. Indian/Alaska Native.

**Table 2:** Clinical and pathological presentation of the study population.

Characteristics	Levels	Total (N = 120,754)	No Metastasis (N = 119,778)	Bone Metastasis (N = 976)	*p*-Value
Presentation					
Histological Type	Papillary	107,227 (88.8)	106,858 (89.2)	369 (37.8)	<0.001
	Follicular	5718 (4.7)	5487 (4.6)	231 (23.7)	
	Anaplastic	649 (0.5)	534 (0.4)	115 (11.8)	
	Medullary	1993 (1.7)	1895 (1.6)	98 (10)	
	Mixed Med	131 (0.1)	126 (0.1)	5 (0.5)	
	Others	5036 (4.2)	4878 (4.1)	158 (16.2)	
T Staging	T1	69,399 (57.5)	69,285 (57.8)	114 (11.7)	<0.001
	T2	20,454 (16.9)	20,342 (17.0)	112 (11.5)	
	T3	17,781 (14.7)	17,607 (14.7)	174 (17.8)	
	T4	4058 (3.4)	3726 (3.1)	332 (34.0)	
N Staging	N0	85,544 (70.8)	85,149 (71.1)	395 (40.5)	<0.001
	N1	29,275 (24.2)	28,830 (24.1)	445 (45.6)	
	NA	5935 (4.9)	5799 (4.8)	136 (13.9)	
Extension to Nearby Structures	No Extension	27,082 (22.4)	26,685 (22.3)	397 (40.7)	<0.001
	Ext into strap muscles	37,440 (31)	37,211 (31.1)	229 (23.5)	
	Ext into the larynx or trachea	56,232 (46.6)	55,882 (46.7)	350 (35.9)	
Management					
Cancer-directed Surgery	Positive	4568 (3.8)	4154 (3.5)	414 (42.5)	<0.001
Radiotherapy	Positive	115,956 (96.2)	115,395 (96.5)	561 (57.5)	
Radioactive iodine	Positive	70,356 (58.3)	70,029 (58.5)	327 (33.5)	<0.001
Systemic therapy	Positive	50,398 (41.7)	49,749 (41.5)	649 (66.5)	

Data are presented as numbers and percentages. A two-sided chi-square test was used. Mixed Med: Mixed medullary thyroid cancer with other thyroid cancer subtypes, N0: lymph node stage with no infiltration, N1: lymph node stage equivalent to lymph node infiltration, NA: not applicable and not defined. Statistical significance was set at *p*-value < 0.05.

**Table 3:** Disease outcomes in thyroid cancer patients with and without bone metastasis.

Characteristics	Levels	Total (N = 120,754)	No Metastasis (N = 119,778)	Bone Metastasis (N = 976)	*p*-Value
Recurrence	Negative	120,354 (99.7)	119,381 (99.7)	973 (99.7)	0.89
	Positive	400 (0.3)	397 (0.3)	3 (0.3)	
Second primary cancers	Negative	98,681 (81.7)	97,927 (81.8)	754 (77.3)	<0.001
	Positive	22,073 (18.3)	21,851 (18.2)	222 (22.7)	
Survival status	Alive	113,335 (93.9)	112,928 (94.3)	407 (41.7)	<0.001
	Dead	7419 (6.1)	6850 (5.7)	569 (58.3)	
Cause of death	Alive	113,334 (93.9)	112,927 (94.3)	407 (41.7)	<0.001
	Dead, other cause	5130 (4.2)	5023 (4.2)	107 (11)	
	Dead, this cancer	2289 (1.9)	1827 (1.5)	462 (47.3)	
Survival time (years)	Median (IQR)	4.25 (1.8–6.8)	4.2 (1.8–6.8)	1.17 (0.25–3.5)	<0.001

Data are presented as numbers and percentages or median and interquartile range (IQR). Two-sided chi-square and Mann–Whitney U tests were used. Statistical significance was set at *p*-value < 0.05.

**Table 4:** Predictor risk factors for second primary cancer following thyroid cancer with and without bone metastasis.

Risk Factor	No Bone Metastasis		Bone Metastasis	
	OR (95%CI)	*p*-Value	OR (95%CI)	*p*-Value
Age ≥ 55 vs. <55 y	3.91 (3.77–4.04)	<0.001	3.64 (1.75–7.57)	0.001
Male vs. Female	1.32 (1.27–1.37)	<0.001	1.09 (0.69–1.72)	0.71
Black vs. White	0.93 (0.87–1.00)	0.046	1.4 (0.77–2.57)	0.27
API vs. White	0.78 (0.73–0.82)	<0.001	0.46 (0.21–0.99)	0.047
AI/AN vs. White	0.85 (0.68–1.07)	0.17	0 (0.00–0.00)	1.00
Urban vs. Rural	1 (0.95–1.06)	0.91	1.65 (0.72–3.79)	0.23
High vs. Low income	1.07 (1.03–1.11)	<0.001	0.95 (0.58–1.55)	0.84
FTC vs. PTC	1 (0.92–1.08)	0.96	1.04 (0.60–1.78)	0.90
ATC vs. PTC	0.74 (0.59–0.93)	0.009	1.32 (0.63–2.76)	0.47
MTC vs. PTC	1.43 (1.27–1.60)	<0.001	1.28 (0.62–2.63)	0.51
N1 vs. N0 stage	0.97 (0.93–1.01)	0.19	0.91 (0.54–1.54)	0.72
Distant node metastasis vs. none	0.59 (0.27–1.28)	0.18	1.21 (0.49–2.99)	0.68
Surgery vs. none	0.41 (0.37–0.46)	<0.001	2.45 (1.26–4.78)	0.008
Radiation vs. none	1 (0.96–1.04)	0.93	0.64 (0.38–1.09)	0.10
Systematic therapy vs. none	1.03 (0.99–1.06)	0.15	0.8 (0.47–1.36)	0.41

Data are presented as odds ratio (OR) and 95% confidence interval (CI). A binary logistic regression model was employed. Significance was set at *p*-value < 0.05. Red values indicate a significant association with increased risk, while values written in blue indicate a significant association with less risk = protective ones. API: Asian or Pacific Islander, AI/AN: Am. Indian/Alaska Native, PTC: papillary thyroid cancer, FTC: follicular thyroid cancer, ATC: anaplastic thyroid cancer, MTC: medullary thyroid cancer.

**Table 5:** Predictor risk factors for second primary cancer following different histopathological thyroid cancer variants.

Risk Factor	PTC		FTC		ATC		MTC	
	OR (95%CI)	*p*-Value	OR (95%CI)	*p*-Value	OR (95%CI)	*p*-Value	OR (95%CI)	*p*-Value
Age ≥ 55 vs. <55 y	3.91 (3.77–4.06)	<0.001	4.14 (3.51–4.88)	<0.001	2.16 (1.01–4.63)	0.048	3.08 (2.44–3.90)	<0.001
Male vs. Female	1.33 (1.28–1.39)	<0.001	1.17 (0.99–1.38)	0.06	0.75 (0.50–1.12)	0.15	1.35 (1.07–1.70)	0.011
Black vs. White	0.96 (0.90–1.04)	0.31	0.73 (0.56–0.95)	0.019	0.75 (0.34–1.65)	0.48	0.85 (0.56–1.30)	0.45
API vs. White	0.78 (0.73–0.82)	<0.001	0.8 (0.60–1.07)	0.13	0.69 (0.35–1.38)	0.29	0.62 (0.38–1.03)	0.06
AI/AN vs. White	0.8 (0.63–1.02)	0.07	1.58 (0.61–4.12)	0.34	---	---	0.98 (0.23–4.12)	0.98
Urban vs. Rural	1.0 (0.94–1.06)	0.95	1.09 (0.85–1.39)	0.50	1.2 (0.68–2.13)	0.52	0.85 (0.59–1.22)	0.38
High vs. Low income	1.07 (1.03–1.11)	0.001	1.07 (0.90–1.27)	0.44	1.01 (0.65–1.56)	0.96	1.07 (0.84–1.37)	0.58
N1 vs. N0 stage	0.97 (0.92–1.01)	0.17	1.2 (0.79–1.83)	0.40	1.14 (0.76–1.71)	0.52	0.9 (0.69–1.17)	0.41
Bone metastasis vs. none	1.28 (0.52–3.14)	0.58	0.58 (0.17–1.98)	0.38	0.5 (0.05–4.94)	0.55	0.47 (0.07–3.17)	0.44
Distant node metastasis vs. none	0.71 (0.31–1.63)	0.41	0.77 (0.08–7.63)	0.82	0.85 (0.29–2.47)	0.76	0.67 (0.13–3.49)	0.63
Multiple organs vs. bone only	0.54 (0.29–1.01)	0.054	1.03 (0.45–2.37)	0.93	1.08 (0.30–3.85)	0.90	1.35 (0.40–4.62)	0.62
Surgery vs. none	0.39 (0.35–0.43)	<0.001	0.73 (0.43–1.25)	0.25	1.19 (0.72–1.99)	0.49	0.99 (0.52–1.88)	0.98
Radiation vs. none	0.99 (0.95–1.03)	0.62	1.04 (0.88–1.22)	0.67	0.72 (0.47–1.10)	0.12	1.99 (1.40–2.84)	<0.001
Systematic therapy vs. none	1.02 (0.99–1.06)	0.22	1.03 (0.87–1.21)	0.72	1.1 (0.65–1.87)	0.71	1.18 (0.94–1.49)	0.15

Data are presented as odds ratio (OR) and 95% confidence interval (CI). A binary logistic regression model was employed. Significance was set at *p*-value <0.05. Red values indicate a significant association with increased risk, while values written in blue indicate a significant association with less risk = protective ones. API: Asian or Pacific Islander, AI/AN: Am. Indian/Alaska Native, PTC: papillary thyroid cancer, FTC: follicular thyroid cancer, ATC: anaplastic thyroid cancer, MTC: medullary thyroid cancer.

**Table 6:** Predictor risk factors for overall survival.

Risk Factor	HR	LL	UL	*p*-Value
**Demographics**				
Age at diagnosis: ≥55 vs. <55 years	5.74	5.35	6.15	<0.001
Sex: Male vs. Female	1.58	1.50	1.67	<0.001
Race: Black vs. White	1.41	1.29	1.55	<0.001
Race: API vs. White	0.94	0.86	1.03	0.19
Race: AI/AN vs. White	1.34	0.95	1.87	0.09
Residency: Urban vs. Rural	0.82	0.75	0.89	<0.001
Income: ≥$75K vs. <$75K	0.85	0.80	0.90	<0.001
**Pathological presentation**				
Follicular vs. Papillary	1.11	0.99	1.25	0.07
Anaplastic vs. Papillary	7.03	6.17	8.02	<0.001
Medullary vs. Papillary	1.36	1.18	1.57	<0.001
T2 vs. T1 stage	1.27	1.17	1.38	<0.001
T3 vs. T1 stage	1.76	1.63	1.89	<0.001
T4 vs. T1 stage	4.48	4.06	4.94	<0.001
N1 vs. N0	1.25	1.17	1.34	<0.001
**Bone metastasis**				
Bone metastasis vs. none	2.78	2.34	3.30	<0.001
Type of concomitant organ metastasis				
1-Brain metastasis vs. Bone only	1.62	1.03	2.55	0.035
2-Liver metastasis vs. Bone only	0.98	0.71	1.34	0.88
3-Lung metastasis vs. Bone only	0.81	0.64	1.02	0.07
Distant LN metastasis vs. none	0.80	0.56	1.15	0.23
**Management**				
Primary cancer surgery vs. none	0.20	0.18	0.22	<0.001
Radiation therapy vs. none	0.66	0.62	0.70	<0.001
Systematic therapy vs. none	0.91	0.86	0.96	0.001

Data are presented as hazards ratio (HR) and 95% confidence interval (CI) for lower limit (LL) and upper limit (UL). The Cox regression model was employed. Significance was set at *p*-values less than 0.05. Red values indicate a significant association with increased risk, while values written in blue indicate a significant association with less risk = protective ones. API: Asian or Pacific Islander, AI/AN: Am. Indian/Alaska Native, PTC: papillary thyroid cancer, FTC: follicular thyroid cancer, ATC: anaplastic thyroid cancer, MTC: medullary thyroid cancer.

## Data Availability

Publicly available datasets were analyzed in this study. These data can be found here: (https://seer.cancer.gov/, accessed on 29 January 2023).

## References

[1] Pstrąg N., Ziemnicka K., Bluyssen H., Wesoły J. (2018). Thyroid cancers of follicular origin in a genomic light: In-depth overview of common and unique molecular marker candidates. Mol. Cancer.

[2] Liu F.C., Lin H.T., Lin S.F., Kuo C.F., Chung T.T., Yu H.P. (2017). Nationwide cohort study on the epidemiology and survival outcomes of thyroid cancer. Oncotarget.

[3] Toraih E.A., Hussein M.H., Zerfaoui M., Attia A.S., Marzouk Ellythy A., Mostafa A., Ruiz E.M.L., Shama M.A., Russell J.O., Randolph G.W. (2021). Site-Specific Metastasis and Survival in Papillary Thyroid Cancer: The Importance of Brain and Multi-Organ Disease. Cancers.

[4] Iñiguez-Ariza N.M., Bible K.C., Clarke B.L. (2020). Bone metastases in thyroid cancer. J. Bone Oncol..

[5] Wu D., Gomes Lima C.J., Moreau S.L., Kulkarni K., Zeymo A., Burman K.D., Wartofsky L., Van Nostrand D. (2019). Improved Survival after Multimodal Approach with ^131^I Treatment in Patients with Bone Metastases Secondary to Differentiated Thyroid Cancer. Thyroid.

[6] Zhang R., Zhang W., Wu C., Jia Q., Chai J., Meng Z., Zheng W., Tan J. (2022). Bone metastases in newly diagnosed patients with thyroid cancer: A large population-based cohort study. Front. Oncol..

[7] Qiu Z.L., Shen C.T., Sun Z.K., Song H.J., Zhang G.Q., Luo Q.Y. (2019). Lung Metastases from Papillary Thyroid Cancer with Persistently Negative Thyroglobulin and Elevated Thyroglobulin Antibody Levels During Radioactive Iodine Treatment and Follow-Up: Long-Term Outcomes and Prognostic Indicators. Front. Endocrinol..

[8] Moneke I., Kaifi J.T., Kloeser R., Samson P., Haager B., Wiesemann S., Diederichs S., Passlick B. (2018). Pulmonary metastasectomy for thyroid cancer as salvage therapy for radioactive iodine-refractory metastases. Eur. J. Cardiothorac. Surg..

[9] Fragnaud H., Mattei J.C., Le Nail L.R., Nguyễn M.V., Schubert T., Griffin A., Wunder J., Biau D., Gouin F., Bonnevialle P. (2022). Mid and long-term overall survival after carcinologic resections of thyroid cancer bone metastases. Front. Surg..

[10] Kondraciuk J.D., Rice S.L., Zhou X., Gharzeddine K., Knezevic A., Spratt D.E., Sabra M., Larson S.M., Grewal R.K., Osborne J.R. (2019). Thyroid Cancer Bone Metastasis: Survival and Genomic Characteristics of a Large Tertiary Care Cohort. Clin. Nucl. Med..

[11] Tong Y., Hu C., Huang Z., Fan Z., Zhu L., Song Y. (2020). Novel nomogram to predict risk of bone metastasis in newly diagnosed thyroid carcinoma: A population-based study. BMC Cancer.

[12] Farooki A., Leung V., Tala H., Tuttle R.M. (2012). Skeletal-related events due to bone metastases from differentiated thyroid cancer. J. Clin. Endocrinol. Metab..

[13] Matta-Coelho C., Simões-Pereira J., Vilar H., Leite V. (2019). Bone Metastases from Thyroid Carcinoma of Follicular Origin: A Single Institutional Experience. Eur. Thyroid J..

[14] Orita Y., Sugitani I., Toda K., Manabe J., Fujimoto Y. (2011). Zoledronic acid in the treatment of bone metastases from differentiated thyroid carcinoma. Thyroid.

[15] Albano D., Bertagna F., Bonacina M., Durmo R., Cerudelli E., Gazzilli M., Panarotto M.B., Formenti A.M., Mazziotti G., Giustina A. (2018). Possible delayed diagnosis and treatment of metastatic differentiated thyroid cancer by adopting the 2015 ATA guidelines. Eur. J. Endocrinol..

[16] Qi L., Zhang W., Ren X., Xu R., Liu C., Tu C., Li Z. (2022). Incidence and Predictors of Synchronous Bone Metastasis in Newly Diagnosed Differentiated Thyroid Cancer: A Real-World Population-Based Study. Front. Surg..

[17] Liu W.C., Li Z.Q., Luo Z.W., Liao W.J., Liu Z.L., Liu J.M. (2021). Machine learning for the prediction of bone metastasis in patients with newly diagnosed thyroid cancer. Cancer Med..

[18] Slook O., Levy S., Slutzky-Shraga I., Tsvetov G., Robenshtok E., Shimon I., Benbassat C., Hirsch D. (2019). Long-Term Outcomes and Prognostic Factors in Patients with Differentiated Thyroid Carcinoma and Bone Metastases. Endocr. Pract..

[19] Vuong H.G., Duong U.N.P., Pham T.Q., Tran H.M., Oishi N., Mochizuki K., Nakazawa T., Hassell L., Katoh R., Kondo T. (2018). Clinicopathological Risk Factors for Distant Metastasis in Differentiated Thyroid Carcinoma: A Meta-Analysis. World J. Surg..

[20] Do M.Y., Rhee Y., Kim D.J., Kim C.S., Nam K.H., Ahn C.W., Cha B.S., Kim K.R., Lee H.C., Park C.S. (2005). Clinical features of bone metastases resulting from thyroid cancer: A review of 28 patients over a 20-year period. Endocr. J..

[21] Wexler J.A. (2011). Approach to the thyroid cancer patient with bone metastases. J. Clin. Endocrinol. Metab..

[22] Marcocci C., Pacini F., Elisei R., Schipani E., Ceccarelli C., Miccoli P., Arganini M., Pinchera A. (1989). Clinical and biologic behavior of bone metastases from differentiated thyroid carcinoma. Surgery.

[23] Wu K., Hou S.M., Huang T.S., Yang R.S. (2008). Thyroid carcinoma with bone metastases: A prognostic factor study. Clin. Med. Oncol..

[24] Mazziotti G., Formenti A.M., Panarotto M.B., Arvat E., Chiti A., Cuocolo A., Dottorini M.E., Durante C., Agate L., Filetti S. (2018). Real-life management and outcome of thyroid carcinoma-related bone metastases: Results from a nationwide multicenter experience. Endocrine.

[25] Choi Y.M., Kim W.G., Kwon H., Jeon M.J., Lee J.J., Ryu J.S., Hong E.G., Kim T.Y., Shong Y.K., Kim W.B. (2016). Early prognostic factors at the time of diagnosis of bone metastasis in patients with bone metastases of differentiated thyroid carcinoma. Eur. J. Endocrinol..

[26] Nervo A., Ragni A., Retta F., Gallo M., Piovesan A., Liberini V., Gatti M., Ricardi U., Deandreis D., Arvat E. (2021). Bone metastases from differentiated thyroid carcinoma: Current knowledge and open issues. J. Endocrinol. Investig..

